# Serum Uric Acid, Alanine Aminotransferase, Hemoglobin and Red Blood Cell Count Levels in Pseudoexfoliation Syndrome

**DOI:** 10.1155/2015/914098

**Published:** 2015-05-13

**Authors:** Hüseyin Simavlı, Yasin Yücel Bucak, Mehmet Tosun, Mesut Erdurmuş

**Affiliations:** ^1^Department of Ophthalmology, School of Medicine, Pamukkale University, 20700 Denizli, Turkey; ^2^Denizli State Hospital, Eye Clinic, 20010 Denizli, Turkey; ^3^Department of Biochemistry, Faculty of Medicine, Abant Izzet Baysal University, 14080 Bolu, Turkey; ^4^Department of Ophthalmology, Faculty of Medicine, Hacettepe University, 06100 Ankara, Turkey

## Abstract

*Purpose*. The pathogenesis of pseudoexfoliation (PEX), the most common cause of secondary glaucoma, has not been clearly identified, but there is increasing evidence that points out the role of oxidative stress. The aim of this study is to evaluate some of the most commonly used blood parameters, hemoglobin (Hb), red blood cell count (RBC), alanine aminotransferase (ALT), and uric acid (UA) levels, in subjects with PEX. *Materials and Methods*. This study is performed in a state hospital between November 2011 and December 2012. Retrospective chart review of subjects who underwent cataract surgery was performed. Thirty-one healthy subjects with PEX and 34 healthy subjects without PEX were evaluated. Hb, RBC, ALT, and UA levels were recorded. Student's *t*-test was used to compare the two groups. *Results*. The mean age was 73.6 ± 14.1 years in PEX group and 70.1 ± 12.7 in control group (*p* = 0.293). Hb, RBC, ALT, and UA levels did not show a statistically significant difference among PEX and control groups (*p* > 0.05 for all). *Conclusion*. Serum levels of Hb, RBC, ALT, and UA levels were similar in subjects with and without PEX. Further studies are needed to clarify the precise role of Hb, RBC, ALT, and UA in the pathogenesis of PEX.

## 1. Introduction

Pseudoexfoliation (PEX) is a late onset disease with an increased prevalence by age. Although it is well known by its ocular features, accumulation of extracellular fibrillar material in the whole body [1] suggests that PEX is a systemic disease. In addition to that, PEX was observed to be a risk factor in systemic diseases such as peripheral vascular diseases [2], renal arterial stenosis [3], and cardiovascular diseases [4]. Despite the fact that the pathogenesis of PEX has not been clearly identified, there is increasing evidence that points out the role of oxidative stress [5–9].

Uric acid (UA) is the end product of purine metabolism. Xanthine oxidase (XO) catalyzes the last two reactions:

(1) $$\begin{array}{c} \text{hypoxanthine}+{\text{H}}_{2}\text{O}+{\text{O}}_{2}⇌\text{xanthine}+{\text{H}}_{2}{\text{O}}_{2}\\ \text{xanthine}+{\text{H}}_{2}\text{O}+{\text{O}}_{2}⇌\text{uric acid}+{\text{H}}_{2}{\text{O}}_{2} \end{array}$$

UA which is one of the most important antioxidants of plasma [10, 11] has been shown to provide up to %65 of total plasma antioxidant capacity [12]. UA can be used as a marker of oxidative stress in hypertension [13] because of the enzyme, XO, which catalyzes the reactions of hypoxanthine to xanthine and xanthine to UA and generates one molecule of hydrogen peroxide in each step. In theory, increased activity of XO in PEX can cause hyperuricemia [14]. Supporting this hypothesis, increased adenosine deaminase activity in PEX, which catalyzes an irreversible reaction in purine catabolism, can be an additional factor in increment of UA [14]. On the other hand, increased oxidative stress in PEX might lead to a decrease in uric acid levels, hypouricemia, because it is one of the most important antioxidants of the serum [6, 9]. In both situations, UA levels may be affected in PEX.

Alanine aminotransferase (ALT) is an enzyme which represents the hepatocellular injury levels. It catalyzes the reversible reaction of

(2) $$\begin{array}{c} \text{L-glutamate}+\text{pyruvate}⇌\alpha \text{-ketoglutarate}+\text{L-alanine} \end{array}$$

Besides this feature, ALT was shown to be an independent marker for oxidative stress [15] and ALT has been reported to correlate with oxidative stress markers [16].

Most of the parameters used in routine blood tests such as complete blood count (CBC) and biochemical parameters have been shown to be related with oxidative stress [15, 17–20]. Hemoglobin (Hb) is an iron containing metalloprotein in red blood cells (RBCs) which is used to carry O_2_ from the lungs to the body and CO_2_ to lungs from the body. Besides this main vital function in the body, RBCs and Hb were reported to reduce the effect of oxidative stress on DNA [17].

All of these parameters mentioned above have a direct or an indirect relationship with oxidative stress. The aim of this study is to reveal if there is a relationship among the routine blood parameters (Hb, RBC, ALT, and UA) and PEX. To the best of our knowledge these parameters have not been studied yet.

## 2. Materials and Methods

The files of patients who underwent cataract surgery in state hospital between November 2011 and December 2012 were evaluated. Patients with any systemic illness (diabetes, hypertension, connective tissue diseases, cardiovascular diseases, stroke, autoimmune disease, renal and liver failure, etc.), drug use, and active smoking were not included in the study. The presence of any ocular diseases except cataract and PEX were not included. All of the patients had a record of complete ophthalmic examination including visual acuity, Goldmann applanation tonometry, slit lamp biomicroscopic examination, and dilated anterior and posterior segment examination. The presence of subjects with and without PEX was recorded. Subjects who had transillumination defects, poor dilatation, and peripapillary atrophy were not included in the study because of the possibility of subtle PEX. Hb, RBC, ALT, and UA values of the patients were recorded from the blood tests performed.

Statistical analysis was made by SPSS 17.00 (SPSS Inc., Chicago, IL, USA). Continuous variables were expressed as mean and standard deviation. The Kolmogorov-Smirnov test was used whether the data distribution were homogeneous. Age and preoperative laboratory tests among the two groups were evaluated by independent samples *t*-test. Chi-square test was used for evaluation of the gender status. A *p* value <0.05 was considered statistically significant.

## 3. Results

There were 31 healthy subjects with PEX and 34 healthy subjects without PEX. All were Caucasian. There were no age and sex differences among the groups. Descriptive values of groups including age and sex were shown in Table 1.

Although serum levels of Hb, RBC, ALT, and UA were all higher in PEX group, the difference was not statistically significant (*p* = 0.346, *p* = 0.805, and *p* = 0.547 and 0.560, resp.). The details were shown in Table 2.

## 4. Discussion

Oxidative stress can be defined as increased intra/extracellular concentrations of reactive oxygen species such as superoxide anion (O_2_^−^), hydrogen peroxide (H_2_O_2_), hydroxyl radical (OH^−^), peroxyl radical, and singlet oxygen (^1^O_2_) over the range of physiologic values. Under normal conditions, ROS are stabilized by antioxidants which can be divided into two sections: enzymatic and nonenzymatic. The most common nonenzymatic antioxidants are glutathione, ascorbic acid, and uric acid. If the increase in oxidative stress cannot be balanced by antioxidants, then oxidants can lead to damage to the lipids (the loss of the integrity of cell membrane), protein denaturation, and DNA mutations, resulting in cell death [21]. Various kinds of diseases like aging, cancer development, and cardiovascular diseases are related to oxidative stress [22]. Moreover, ocular diseases like cataract and glaucoma are associated with oxidative stress [14]. Furthermore, growing evidence supports the importance of oxidative stress in PEX [5–9, 14]. Understanding the role of antioxidants in the development of PEX can provide different approaches for improved therapeutic intervention and screening.

PEX is a systemic disease in which oxidative stress plays an important role in the pathogenesis of the disease. Total antioxidant status [23], antioxidants like ascorbic acid [24] or methionine [6], trace elements such as zinc [25] or selenium [26], and antioxidative enzymes such as superoxide dismutase [27] and glutathione peroxidase [27] were all decreased in PEX syndrome regardless of the samples taken from serum, aqueous humor, or tissue. Likewise, XO, a prooxidant enzyme, has been shown to be increased in serum [14]. On the other hand, total oxidant status [23] and inhibitors of the antioxidant enzymes such as asymmetric dimethyl arginine were increased [28]. In general, antioxidants are decreased and total oxidant status is increased in PEX.

Uric acid (UA) is one of the most important antioxidants of plasma [10, 11]. It is the end product of purine nucleotides. It scavenges reactive oxygen species (ROS) and protects erythrocyte membrane integrity [29]. It is higher than the other nonenzymatic antioxidants like ascorbate, glutathione, tocopherols, and methionine [30]. UA is present in all extracellular spaces like plasma, synovial space, amniotic fluid, aqueous humor, and the vitreous body [30, 31]. Likewise, Wayner et al. [12] showed that urate provides up to %65 of total plasma antioxidant capacity. UA level was shown to be related to cardiovascular diseases [32], hypertension [33] and diabetes mellitus [34]. Increased UA serum levels in normotension glaucoma [35], as an indicator of imbalance in oxidant/antioxidants, have been reported. As far as we know, this is the first study which evaluates UA in PEX. There was no statistically significant difference found in serum UA levels of subjects with or without PEX (Table 2). Smoking, obesity, and dietary factors were also reported to alter uric acid levels [36]. Although we checked out smoking in our patients, we did not determine obesity or dietary factors in our study because of lack of detailed information in patients' files. XO, which produces UA, was reported to be increased in PEX [14]. So an increased UA level can be expected in PEX. But, on the other hand, an increased total oxidative stress in PEX can cause a decrease in UA levels. We think that there may be a balance in UA. Increased oxidative stress may cause a decrease in UA which is balanced by UA production by XO.

Although the literature is contradictive, RBCs, especially Hb in RBCs, may have an effect on DNA repair. Gafter-Gvili et al. [17] represented that Hb decreased oxidative stress related DNA damage. Supporting this, Hb protected lymphocytes against the chromosome breaks caused by Diepoxybutane, an alkylating agent [37]. On the other hand, Aksu et al. [38] reported that there was no association between DNA damage and Hb. Additionally, some authors have reported contradictory results in which Hb caused DNA damage in the same fashion of H_2_O_2_ [39, 40]. In our study, although Hb levels and RBCs count were higher in PEX, these differences were not statistically significant (*p* = 0.346, *p* = 0.805).

While we found an increased ALT level in PEX, the difference was not statistically significant (*p* = 0.547). PEX has been reported to accumulate in liver, but it was not reported to cause liver dysfunction [1].

Although PEX has been reported with an increased oxidative stress, we did not find a significant difference in Hb, RBC, ALT, and UA levels. Because the pathogenesis of PEX has not been clearly identified, increased oxidative stress and alterations in antioxidant system do not always necessitate an alteration of all the enzymes related with oxidative system.

One of the limitations of this study is the design of the study. This retrospective study can be a pilot study for upcoming prospective studies. Another limitation is the small sample size which can affect the results.

In conclusion, UA, ALT, and Hb levels and RBCs count were not changed in subjects with PEX. Further prospective studies are needed to clarify the precise role of UA and antioxidants in the pathogenesis of PEX.

## Figures and Tables

**Table 1:** Demographics of pseudoexfoliation and control groups.

	PEX group Mean ± SD	Control group Mean ± SD	*p*
Age (years)	73.6 ± 14.1	70.1 ± 12.7	0.293
Sex (male/female)	15/16	17/17	0.920

PEX: pseudoexfoliation; SD: standard deviation.

**Table 2:** Serum uric acid, alanine aminotransferase, hemoglobin levels, and red blood cell count of pseudoexfoliation (*n* = 31) and control groups (*n* = 34).

	Reference range	PEX group Mean ± SD	Control group Mean ± SD	*p*
Uric acid (mg/mL)	3.4–7.0	5.27 ± 1.22	5.07 ± 1.54	0.560
ALT (U/L)	1–40	14.15 ± 5.57	13.87 ± 7.48	0.547
HGB (g/dL)	11.5–17.5	13.83 ± 0.67	13.51 ± 0.96	0.346
RBC (M/uL)	3.90–5.50	4.61 ± 0.40	4.56 ± 0.45	0.805

PEX: pseudoexfoliation, SD: standard deviation, HGB: hemoglobin, RBC: red blood cell count, and ALT: alanine aminotransferase.
